# Costs, Coverage, and Acceptability of Azithromycin Mass Administration to Children 1–11 Versus 1–59 Months Old to Reduce Mortality: A Cluster-Randomized Trial in Niger

**DOI:** 10.4269/ajtmh.24-0723

**Published:** 2025-04-08

**Authors:** Ahmed M. Arzika, Ramatou Maliki, Abdou Amza, Alio Karamba, Nasser Gallo, Bawa Aichatou, Ismael I. Sara, Diallo Beidi, Laminou M. Haroun, Farissatou Oumarou, Carolyn Brandt, Brittany Peterson, Elodie Lebas, Emily Colby, William Nguyen, Zijun Liu, Benjamin F. Arnold, Thomas M. Lietman, Meagan C. Fitzpatrick, Kieran S. O’Brien

**Affiliations:** ^1^ Centre de Recherche et Interventions en Santé Publique, Birni N’Gaoure, Niger;; ^2^ Programme Nationale de Santé Oculaire, Niamey, Niger;; ^3^ Francis I. Proctor Foundation, University of California, San Francisco, California;; ^4^ Department of Ophthalmology, University of California, San Francisco, California;; ^5^ Institute for Global Health Sciences, University of California, San Francisco, California;; ^6^ Department of Epidemiology and Biostatistics, University of California, San Francisco, California;; ^7^ Center for Vaccine Development and Global Health, University of Maryland School of Medicine, Baltimore, Maryland

## Abstract

Azithromycin mass drug administration (MDA) for 1- to 59-month-olds reduces child mortality. However, guidelines restrict eligibility to 1- to 11-month-olds because of concerns about antimicrobial resistance. This cluster-randomized implementation trial was conducted in parallel with a larger efficacy trial and compared implementation outcomes between these approaches. Rural communities in Niger were randomly assigned to receive biannual azithromycin MDA for either 1- to 59-month-olds or 1- to 11-month-olds over 1 year. The primary outcome was the community-level cost per dose delivered. Secondary outcomes included reach (coverage), as well as acceptability, appropriateness, and feasibility according to participants and providers. In November 2020, 40 eligible communities were randomly assigned to each arm, with 37 communities in the 1- to 59-month arm and 39 communities in the 1- to 11-month arm contributing to analyses. The mean cost per dose delivered was $6.50 lower (95% CI –$10.40 to –$3.70; *P*-value <0.001) in the 1- to 59-month arm ($1.60; 95% CI $1.00 to $2.30) compared with the 1- to 11-month arm ($8.20; 95% CI $7.60 to $8.80). Treatment coverage was similar by arm and exceeded 90% in both distributions. More caregivers in the 1- to 59-month arm found the intervention acceptable (mean difference 4.2%; 95% CI 0 to 8.4%; *P*-value 0.04) and appropriate (3.4%; 95% CI 0.1 to 6.8%; *P*-value 0.04) compared with the 1- to 11-month arm. When combining arms, all groups indicated that including 1- to 59-month-olds was more acceptable, appropriate, and feasible than restricting to 1- to 11-month-olds. No serious adverse events were reported. Overall, including 1- to 59-month-olds resulted in a lower cost per dose delivered than restricting to 1- to 11-month-olds. Community groups perceived both interventions to be acceptable, appropriate, and feasible, but they strongly preferred the 1- to 59-month treatment.

## INTRODUCTION

Azithromycin mass drug administration (MDA) has been shown to reduce child mortality. Several cluster-randomized trials in sub-Saharan African countries have found that biannual distribution of oral azithromycin to children aged 1–59 months reduced mortality by 14–18% over 2 years.^1^^–^^3^ The mechanism of effect is thought to be broad and includes both direct and indirect effects,^3^ with evidence for reductions in respiratory, diarrheal, and malarial disease,^4^^–^^7^ which are the top causes of mortality in sub-Saharan Africa.^8^ As these distributions also increase macrolide resistance,^9^^–^^13^ the WHO guidelines on this intervention recommend restricting azithromycin MDA for child survival to children aged 1–11 months.^14^ Although limiting this intervention may reduce resistance, this approach had not been tested for efficacy at the time of the guidelines’ release and would also result in fewer overall deaths averted.^15^

To provide more evidence on the optimal intervention, the *Azithromycine pour la Vie des Enfants au Niger: Implementation et Recherche* (AVENIR) cluster-randomized trial was designed to compare the impact of targeting children aged 1–59 months versus those aged 1–11 months on mortality and antimicrobial resistance.^16^ These two approaches may also have different effects on implementation outcomes, such as program costs, treatment coverage, and acceptability among caregivers, community health workers (CHWs), and health center leaders, each of which has important consequences for program feasibility. A comparison of implementation outcomes, in addition to mortality and resistance, provides comprehensive information to countries and programs considering this intervention. Here, we used implementation science frameworks and a separate cluster-randomized trial design to compare costs, coverage, and acceptability of azithromycin MDA targeted to children aged 1–11 months versus those aged 1–59 months in a programmatic setting in Niger.^18^^–^^20^ This trial was conducted in parallel to the main AVENIR trial, which focused on mortality and antimicrobial resistance outcomes. A cluster-randomized design was used because of the community-based nature of the MDA intervention and the relevance of community-level outcomes to programs.

## MATERIALS AND METHODS

### Study design.

The AVENIR Delivery II trial was a cluster-randomized trial conducted in the Dosso Region of Niger, in parallel with the main AVENIR trial and another implementation trial in different communities.^3^^,^^21^ Eighty communities were randomized to receive 1 year of biannual azithromycin MDA for children aged 1–59 months or for children aged 1–11 months, and program costs, treatment coverage, and acceptability were compared. The randomization unit was the *grappe*, an administrative unit referred to here as “community.” This trial was conducted concurrently with a larger effectiveness trial and another implementation trial in the same area.^16^^,^^21^

### Participants.

Communities were randomly selected from a larger pool of eligible communities to ensure the generalizability of results across trials. Eligibility criteria for all trials included a population size between 250 and 2,499 people, according to the most recent national census, a distance greater than 5 kilometers from the nearest district headquarters town based on data from *Centres de Santé Integrés* (CSIs), and verbal consent from community leaders. Communities designated as urban in the national census or experiencing insecurity were excluded. None of the included communities had received azithromycin MDA as part of the trachoma program or other child mortality studies in at least the past 10 years. At the individual level, children whose caregivers provided consent were eligible for inclusion, with the eligible age depending on the community randomization assignment (1–59 months or 1–11 months).

### Randomization and masking.

From the eligible communities in the Dosso Region, 80 were randomly selected for inclusion in this trial. The study biostatistician prepared the randomization sequence in R (R Foundation for Statistical Computing, Vienna, Austria). Included communities were randomized in a 1:1 ratio with no stratification or matching. The randomization allocation was implemented by the study team based in Niger. Given the nature of the interventions, participants, investigators, and those implementing the intervention were not masked. Outcome assessors who conducted the post-distribution survey were not actively informed of the intervention but were not completely masked because responses to the survey questions had the potential to reveal allocation. The data analyst was masked to allocation during the initial analyses.

### Procedures.

Two main activities took place before MDA: a predistribution census and sensitization campaigns. The predistribution census was conducted by trained data collectors in the included communities to record the number of eligible children within 1 month of the first MDA. Households were visited door-to-door, and basic demographic data were recorded on heads of households, guardians, and children under 5 living in the household. Data were recorded electronically on a mobile application using CommCare (Dimagi, Cambridge, MA). All communities received a sensitization campaign before distributions. Study team members, district health communications personnel, and CSI leaders participated. Sensitization included a community meeting at the home of each community leader to present the purpose and nature of the study and interventions and to answer questions. Communities were informed of the distribution date 1 day before it occurred by the CSI leader.

All communities received two mass administrations of a single dose of oral azithromycin to eligible children approximately 6 months apart over a 1-year period (referred to as “MDA 1” and “MDA 2” hereafter). Eligible children were 1–59 months old at the time of treatment in the communities randomly assigned to the 1- to 59-month treatment, and 1–11 months old at the time of treatment in communities randomly assigned to the 1- to 11-month treatment. Unlike the full-time study team that delivered the intervention in the main AVENIR trial, the AVENIR Delivery II trial worked within the Ministry of Health system for similar campaigns. The intervention was delivered by existing CHWs (*relais communautaires*) to mimic future program delivery. Community health workers in this area live in or near the communities they serve and support a range of maternal and child health activities. They are overseen by CSIs (primary health care centers). The study team trained CSI leaders to conduct the interventions, and the CSI leaders trained the CHWs before each MDA. Mass drug administration 1 required involvement from higher-level Ministry of Health officials to launch the program. The 1-day trainings included lectures and hands-on practice with the study procedures (determining eligibility, obtaining consent, determining dose, administering the intervention, and completing the paper data collection form). Both the study team and CSI leaders conducted supervision visits to monitor the implementation of the interventions.

Two CHWs participated in the distributions for each community. They were asked to complete each community’s distribution within 2 days, although they could work as many days as necessary to cover the community and were paid for the actual days worked. Community health workers visited every household in each community to identify eligible children for inclusion and to obtain informed consent from caregivers for their participation.

Azithromycin (Zithromax) powder for oral suspension was reconstituted using bottled water. Each dose was measured into a new dosing cup or syringe for children too young to drink from a dosing cup, with a target dose of 20 mg/kg of body weight, up to the adult dose of 1 g. Two-tiered age-based dosing was used for children aged 1–11 months.^22^ Height-based dosing was used for children aged 12–59 months using the dosing tape included in Niger’s trachoma program. Community health workers recorded the number of doses administered by sex and age category (1–11 months or 12–59 months), reasons for ineligibility or refusal, adverse events, and the number of days required for distribution. Data were recorded on a paper form that was later entered electronically by CSI leaders. Caregivers were also asked to inform the CHW about any adverse events experienced by the treated child within 1 month of the distribution. Community health workers reported adverse events to the study team by phone, and serious adverse events were reported by email to Pfizer and the medical monitors based in Niger and the United States.

### Outcomes.

Outcomes were defined at the community level using the Proctor Outcomes for Implementation Research and reach, effectiveness, adoption, implementation, maintenance frameworks.^18^^–^^20^ The primary outcome was the cost per dose delivered at MDA 2. Mass drug administration 2 was selected as the time point for the primary analysis because previous evidence indicates that start-up costs increase the costs associated with the first distribution, and the goal of this analysis was to represent ongoing rather than start-up costs.^21^ Micro-costing was used to estimate program costs, using known unit costs for personnel, training, equipment, and supplies required to distribute azithromycin at the community level.^21^ A standardized comprehensive template was developed, and interviews with staff were conducted to determine unit costs. Community-level costs were validated by comparing micro-costing estimates with routine administrative forms used to track expenditures for budget reporting. Doses delivered and the number of days worked were recorded by CHWs on paper data collection forms, as described above. Costs deemed strictly for research purposes were excluded, as was the cost of azithromycin, which was donated for the study. Costs are reported in 2021 USD using an exchange rate of 550 West African CFA francs per USD to align with related work.^21^ As secondary outcomes, we also calculated the total cost per community, along with delivery- and training-specific costs for each outcome and MDA.

Other secondary outcomes included reach (treatment coverage), acceptability, appropriateness, and feasibility. Reach was defined as the number of doses delivered divided by the estimated number of eligible children, as recorded on the paper data collection forms. The outcome was compared by arm at MDA 2, and estimates are presented for both MDAs. As sensitivity analyses, we also estimated the denominator using the predistribution census of eligible children and estimated caregiver-reported treatment coverage during a post-distribution survey, both of which were available for MDA 1. Acceptability (defined as affective attitude), appropriateness, and feasibility were measured for each arm during the post-distribution survey conducted after MDA 1 among CHWs, community health center leaders, and caregivers.^18^^,^^23^ CHW and caregiver respondents in both arms were also asked whether they perceived an intervention including only children aged 1–11 months or all children aged 1–59 months as more acceptable, appropriate, and feasible. For all community groups, the survey included additional questions on knowledge of the purpose of the intervention (acceptability defined as coherence, referred to as “knowledge of purpose of intervention” elsewhere).^23^ The CHW and health center leader surveys included questions about difficulties encountered during the distribution. The caregiver survey included questions about experiences of adverse events and the perceived relative priority of this intervention compared with other child health interventions, using a five-point scale, with a score of five indicating the most important intervention. Survey instruments were developed in English and then translated into French (Supplemental Information). The French translations were reviewed as a group by study team members fluent in English and French, as well as local languages, including Zarma, Hausa, and Peul. The final wording for each question was determined by group consensus. To assess acceptability, appropriateness, and feasibility, the English and French translations of the acceptability of intervention measure, intervention appropriateness measure, and feasibility of intervention measure validated instruments were reviewed by the group to establish the appropriate wording in each language for the single question used to reflect the domain.^24^ Surveys were conducted within one month of the second MDA by trained data collectors in a door-to-door manner, with data recorded using a mobile application (CommCare). For the caregiver survey, all households in the included communities were visited door-to-door. All CHWs and CSI leaders participating in the trial were included in the surveys.

## STATISTICAL ANALYSES

The inclusion of 80 communities (40 per arm) was anticipated to provide 80% power to detect a difference in cost per dose delivered between arms of $1.09, assuming an alpha of 0.05 and a mean cost per dose delivered of $2.67, with a standard deviation of $1.71 in the 1- to 59-month arm based on previous data.^21^

The primary analysis was prespecified as a comparison of community-level cost per dose delivered between arms, using a permutation test based on a two-sample *t*-test that compared cluster-level means. Cost variables were log-transformed for comparisons because of skewness, and geometric mean costs are presented by arm. Sensitivity analyses included a similar comparison based on the Wilcoxon rank sum test statistic, as well as a linear regression model to compare community-level cost per dose delivered by arm, with a covariate to account for community population size. An additional sensitivity analysis examined costs assuming a minimum of 2 days worked by CHWs for each MDA because some programs in Niger use this approach. Secondary outcomes were analyzed similarly at the community level using permutation tests based on the t-statistic. An alpha of 0.05 was used to determine statistical significance, and 95% CIs were estimated with bootstrapping at the community level.

### Patient and public involvement.

Patients or the public were not involved in the design of this study. The Niger Ministry of Health participated in the study design. The Niger Ministry of Health, CSI leaders, CHWs, and community leaders were informed of the design and were involved in the recruitment, conduct, and dissemination of the study.

## RESULTS

**Figure 1.**
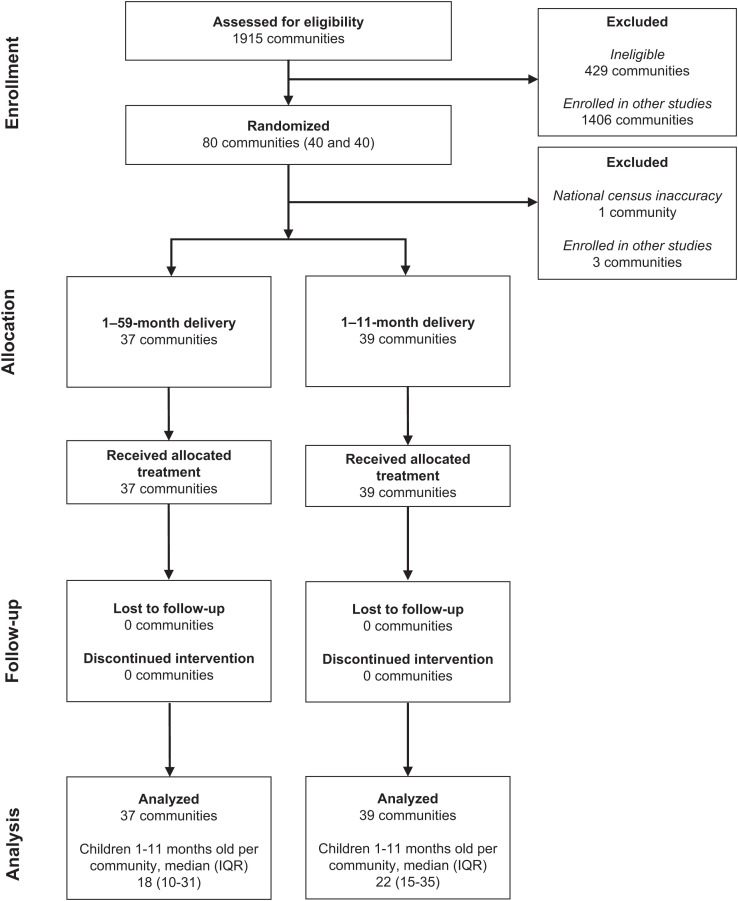
Trial profile. Consolidated Standards of Reporting Trials participant flow diagram.

In November 2020, 1,915 communities in the Dosso Region were screened for inclusion in the AVENIR trials (Figure 1). Eighty eligible communities were randomly selected for inclusion in the AVENIR Delivery II trial and were randomly assigned to receive biannual azithromycin MDA targeted at children aged 1–59 months or 1–11 months over 1 year (from November 9, 2022 to July 4, 2023). After randomization, four communities were excluded because of inaccuracies in national census data or concurrent participation in other similar studies. Ultimately, 37 communities from the 1- to 59-month arm and 39 communities from the 1- to 11-month arm contributed to the analyses.

Baseline characteristics of the included communities were similar across arms (Table 1). Overall, communities had a median total population of 509 (interquartile range [IQR] 387 to 896), according to administrative estimates reported by CSIs. On average, communities were located 27.0 km (SD 10.9) from the nearest CSI, and the estimated median population of children aged 1–59 months per community was 46.0 (IQR 20.5 to 98.8). Treatment coverage was high (>90%) in both arms and MDAs, with no differences demonstrated between arms (Supplemental Table 1). The sensitivity analysis with pretreatment census coverage found similar results (Supplemental Table 1). Caregiver-reported coverage during the MDA 1 survey was lower in both arms compared with CHW-reported coverage and was significantly higher in the 1- to 59-month arm compared with the 1- to 11-month arm (Supplemental Table 1).

**Table 1:** Baseline characteristics of included communities by arm

Characteristic	Azithro 1–59 (*n* = 37)	Azithro 1–11 (*n* = 39)	Overall (*N* = 76)
Total population, n*	55,097	50,316	105,413
Median overall population size (IQR)*	554 (389–896)	500 (361.5–875)	509 (387–896)
Median 1- to 11-month population size (IQR)^†^	18.0 (10.0–30.5)	22.0 (15.0–35.0)	20.0 (13.0–34.0)
Mean distance to nearest district headquarters town in km (SD)*	26.6 (10.7)	27.1 (11.2)	27.0 (10.9)
Mean distance to CSI in km (SD)*	10.1 (9.2)	9.3 (6.7)	9.7 (8.0)

CSI = Centre de Santé Integré (primary health center); IQR = interquartile range.

* Population and distance estimates as reported by CSIs.

^†^ Population of children 1–59 months old was estimated based on a predistribution census.

The primary analysis included 37 communities from the 1- to 59-month arm and 39 communities from the 1- to 11-month arm that received azithromycin during MDA 2 (Table 2). In this round, CHWs in the 1- to 59-month arm delivered a total of 5,827 doses of azithromycin, compared with 1,002 doses in the 1- to 11-month arm. Mass drug administration in the 1- to 59-month arm required an average of 1.4 (SD 0.6) days per community, compared with 1.0 (SD 0.0) days per community in the 1- to 11-month arm (mean difference 0.3 days; 95% CI 0.1 to 0.5; *P*-value 0.0003). The community-level geometric mean cost per dose delivered was $6.50 lower (95% CI –$10.4 to –$3.7; *P*-value <0.001) in the 1- to 59-month arm ($1.60 per dose delivered; 95% CI $1.0 to $2.3) compared with the 1- to 11-month arm ($8.20 per dose delivered; 95% CI $7.60 to $8.80). Sensitivity analyses produced similar results (Supplemental Tables 2–3). The geometric mean cost of delivery and training per community was $20.80 higher (95% CI $12.20 to $29.30; *P*-value <0.001) in the 1- to 59-month arm ($197.30 per community; 95% CI $197.00 to $197.70) compared with the 1- to 11-month arm ($176.50 per community; 95% CI $176.20 to $176.90). A detailed breakdown of costs contributing to the delivery and training categories is provided in Supplemental Table 4.

**Table 2:** Estimates of overall costs and costs per dose delivered by treatment arm at the community level

Variable	MDA 1		MDA 2		Mean Difference in MDA 2* (95% CI)	*P*-Value*
	Azithro 1–59 (*n* = 37)	Azithro 1–11 (*n* = 39)	Azithro 1–59 (*n* = 37)	Azithro 1–11 (*n* = 39)		
Number of days worked by CHWs (mean, SD)	1.4 (0.6)	1.0 (0.0)	1.4 (0.5)	1.0 (0.2)	0.3 (0.1 to 0.5)	0.0003
Overall costs and doses delivered						
Delivery cost (geometric mean, 95% CI)	$113.10 ($112.80 to $113.50)	$92.40 ($92.10 to $92.80)	$115.20 ($114.80 to $115.60)	$95.20 ($94.90 to $95.60)	$20.00 ($12.70 to $29.80)	<0.0001
Training cost (geometric mean, 95% CI)	$136.80 ($136.40 to $137.10)	$143.90 ($143.60 to $144.30)	$81.50 ($81.20 to $81.80)	$81.20 ($80.90 to $81.60)	$0.30 (N/A)^†^	<0.0001
Delivery + training cost (geometric mean, 95% CI)	$250.50 ($250.20 to $250.90)	$236.40 ($236.10 to $236.70)	$197.30 ($197.00 to $197.70)	$176.50 ($176.20 to $176.90)	$20.80 ($12.20 to $29.30)	<0.0001
Doses delivered (geometric mean, SD)	126.17 (85.5)	24.6 (14.0)	157.5 (119.7)	25.7 (15.3)	131.8 (91.6 to 172.0)	<0.0001
Cost per dose delivered						
Delivery cost (geometric mean, 95% CI)	$1.10 ($0.50 to $1.70)	$4.40 ($3.80 to $5.00)	$1.00 ($0.30 to $1.60)	$4.40 ($3.80 to $5.00)	–$3.50 (–$5.50 to –$2.50)	<0.0001
Training cost (geometric mean, 95% CI)	$1.30 ($0.70 to $2.00)	$6.80 ($6.30 to $7.40)	$0.70 ($0.00 to $1.40)	$3.80 ($3.20 to $4.40)	–$3.10 (–$4.80 to –$1.20)	<0.0001
Delivery + training cost (geometric mean, 95% CI)	$2.40 ($1.80 to $3.10)	$11.20 ($10.70 to $11.80)	$1.60 ($1.00 to $2.30)	$8.20 ($7.60 to $8.80)	–$6.50 (–$10.40 to –$3.70)	<0.0001

CHWs = community health workers; MDA = mass drug administration.

* The 1- to 59-month arm compared with the 1- to 11-month arm during MDA 2.

^†^ Too little variability in training costs by arm for comparison.

**Figure 2.**
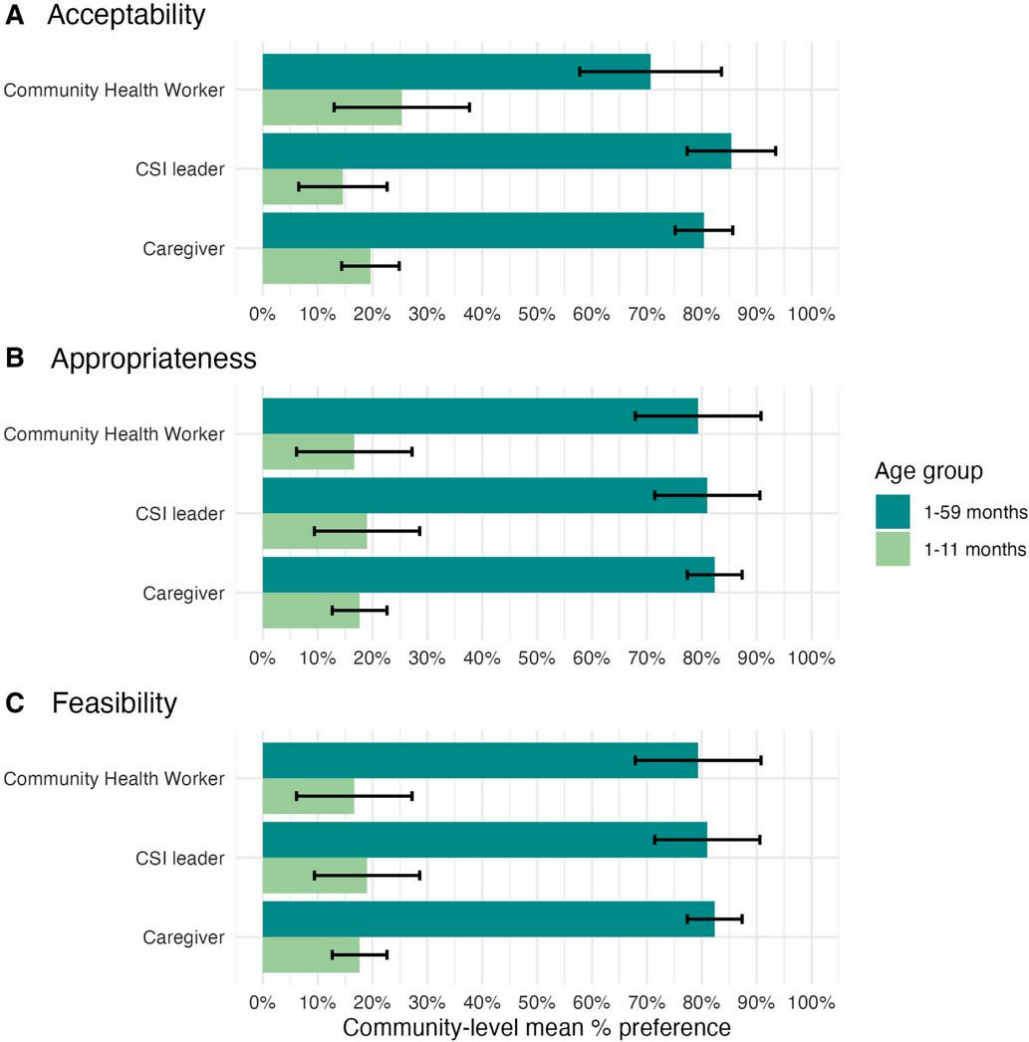
Overall perceptions of acceptability, appropriateness, and feasibility of 1- to 59-month versus 1- to 11-month treatment among community health workers, Centres de Santé Integrés leaders, and caregivers. Note that this figure does not display a by-arm comparison; rather, this figure shows the stated preference for 1- to 59-month or 1- to 11-month treatment by community group in both arms combined. Supplemental Table 9 provides additional details.

The post-distribution survey after MDA 1 included CSI leaders, CHWs, and caregivers of eligible children. Of the 54 CSIs visited, 50 CSI leaders completed the survey (92.6%). Of the 302 CHWs who participated in the MDA, 179 completed the survey (59.3%). Of the 5,016 households visited, 4,931 consented to participate, and 3,429 caregivers participated in the survey (68.4%). Overall, most respondents in each community group reported understanding the purpose of the MDA in their community, with no differences demonstrated by arm (Supplemental Tables 5–9). More caregivers in the 1- to 59-month arm indicated that the intervention was acceptable (mean difference 4.2%; 95% CI 0.0 to 8.4%; *P*-value 0.04) and appropriate (mean difference 3.4%; 95% CI 0.1 to 6.8%; *P*-value 0.04) for their household compared with those in the 1- to 11-month arm, although overall acceptability, appropriateness, and feasibility were high in both arms among all community groups (Supplemental Tables 5–7). When asked whether a 1- to 59-month or a 1- to 11-month delivery was preferable, all community groups in both arms indicated that the 1- to 59-month MDA was more acceptable, appropriate, and feasible (Figure 2; Supplemental Table 9). Caregivers perceived this intervention as a high priority, with an overall mean rank of 4.0 (95% CI 3.8 to 4.1) in the 1- to 59-month arm and 4.1 (95% CI 3.9 to 4.3) in the 1- to 11-month arm on a five-point Likert scale (mean difference –0.1; 95% CI –0.3 to 0.1; *P*-value 0.26; Supplemental Table 7). Community health workers reported that challenges encountered during the distribution largely involved caregivers requesting the inclusion of ineligible children in the MDA, including those from non-target communities and those outside of the target age range (Supplemental Table 5).

No serious adverse events were reported (Table 3). The most common non-serious adverse events reported by caregivers included diarrhea, fever, and vomiting. The overall proportion of caregivers reporting adverse events was similar across arms at the community level, with 5.6% (95% CI 2.5 to 8.7%) in the 1- to 59-month arm and 10.4% (95% CI 5.8 to 15.0%) in the 1- to 11-month arm (mean difference –4.8%; 95% CI –10.3 to 0.7%; *P*-value 0.09).

**Table 3:** Caregiver-reported adverse events after treatment by treatment arm at the community level

Survey Question	Azithro 1–59 (*n* = 37), Mean % Responding “Yes” (95% CI)	Azithro 1–11 (*n* = 39), Mean % Responding “Yes” (95% CI)	Mean Difference* (95% CI)	*P*-Value*
Did your child experience symptoms after treatment?^†^^,^^‡^	5.6% (3.7% to 10.1%)	10.4% (6.7% to 15.6%)	−4.8% (–10.3% to 0.7%)	0.09
Fever	1.0% (0.5% to 1.8%)	2.6% (1.4% to 4.5%)	−1.7% (–3.4% to 0.0%)	0.06
Diarrhea	3.7% (2.1% to 7.5%)	8.3% (5.0% to 13.6%)	−4.6% (–9.5% to 0.4%)	0.08
Vomiting	0.7% (0.4% to 1.2%)	1.8% (0.7% to 3.4%)	−1.0% (–2.4% to 0.4%)	0.16
Abdominal pain	0.5% (0.0% to 2.5%)	0.1% (0.0% to 0.7%)	0.4% (–0.5% to 1.2%)	0.49
None	94.4% (89.9% to 96.3%)	89.6% (84.4% to 93.3%)	4.8% (–0.7% to 10.3%)	0.09

* The 1- to 59-month arm compared with the 1- to 11-month arm during the post-MDA 1 survey.

^†^ Caregiver could have reported more than one symptom.

^‡^ A total of <0.2% of caregivers in each arm reported rash, constipation, or other symptoms.

## DISCUSSION

Although existing evidence of the efficacy of azithromycin MDA to reduce child mortality comes from studies treating children aged 1–59 months,^1^^,^^25^ the WHO guidelines on this intervention recommend treating children aged 1–11 months out of concern for increasing antimicrobial resistance.^14^ The main AVENIR trial compared the impact of these two approaches on mortality and resistance.^16^^,^^17^ The AVENIR Delivery II trial presented here compares the impact of these two approaches on a range of implementation outcomes, including costs, reach, and acceptability. Overall, we found that including children aged 1–59 months with azithromycin MDA resulted in a cost per dose delivered that was $6.50 lower than restricting treatment to children aged 1–11 months. The two approaches resulted in similarly high reach, acceptability, appropriateness, and feasibility, though community groups overwhelmingly preferred the 1- to 59-month intervention.

Program costs are a key driver of feasibility and sustainability as an intervention moves into programmatic implementation. At the community level, the overall cost of azithromycin MDA per community was $20.80 higher with the 1- to 59-month intervention compared with the 1- to 11-month approach. Because training costs are fixed and therefore similar in the two approaches, this difference was largely driven by the delivery to children aged 1–59 months, which required, on average, more days of distribution compared with children aged 1–11 months. However, as the total number of doses delivered was nearly six times larger in the 1- to 59-month arm compared with the 1- to 11-month arm, the mean cost per dose delivered was $6.50 lower. In a sensitivity analysis assuming a fixed minimum number of days for distribution, the overall cost was $14.60 higher with the 1- to 59-month intervention, but the mean cost per dose delivered was still $7.30 lower. Note that azithromycin was donated for this study and is currently being donated for child survival programs using azithromycin MDA. The cost considerations presented here do not include the cost of the drug itself, although future programs may need to account for the cost of azithromycin if donation programs are not continued.

The per-dose cost of azithromycin MDA presented here may be high compared with expectations. A recent study in the same setting found a similar cost per dose delivered of ∼$2 for administering azithromycin MDA to children aged 1–59 months with existing CHWs,^21^ whereas an older 2016 study reported a cost per dose delivered ranging from $0.63 to $0.94 for treating children aged 1–59 months in Malawi.^26^ Mass drug administration is widely used in neglected tropical disease programs and has been associated with significant economies of scale. A systematic review found that MDA programs cost less than $0.50 per dose delivered in 2015 when treating 100,000 people or more, but more than $2.00 per dose delivered when treating fewer than 100,000 people, with unit costs decreasing as the number of implementation years increases.^27^ Given that these are initial treatment rounds on a small scale, our cost estimates for treating children aged 1–59 months are aligned with the systematic review. We anticipate that the cost per child treated will decrease when all communities within eligible CSI catchment areas are included in more rounds of distribution. Indeed, costs decreased between MDA 1 and 2 in this study. In addition, these analyses applied the 2021 currency exchange rate to facilitate comparison with our previous work.^21^ This rate is relatively favorable for CFA, raising the USD cost by ∼10% compared with 2024 exchange rates or the decade average. Finally, the costs presented in the systematic review were mostly from programs relying on volunteers, which decreases costs compared with the program implemented here that paid CHWs. Future work will explore the cost-effectiveness of these two approaches, which will be an important complement to these results because the 1- to 59-month approach is expected to notably increase the number of deaths averted.^15^

Overall, program reach was quite high, with treatment coverage averaging greater than 90% in both arms and rounds of distribution, similar to other programmatic coverage reported for this intervention in the region.^21^^,^^28^ Caregiver-reported coverage was lower, perhaps because of CHWs missing households or overreporting distributions, or potentially reflecting the fact that children from surrounding communities not directly included in this study also presented for treatment and were counted in CHW-based coverage estimates. Mass drug administration programs report a wide range of coverage, with more controlled studies tending to report higher coverage on average than program settings.^1^^,^^21^^,^^29^^–^^32^ Here, we attempted to emulate programmatic implementation by involving the CSI leaders and CHWs, although the presence of the study team for training, supervision, and data collection activities may have resulted in higher participation overall. Campaign-style interventions for child health, such as MDA, are commonly implemented in this setting; therefore, the high reported acceptability, appropriateness, and feasibility of this intervention among community groups was anticipated. Importantly, community groups expressed a clear preference for the treatment for 1- to 59-month-olds treatment compared with the treatment for 1- to 11-month-olds, likely because most child health interventions target this broader age range, leading communities to struggle with the age restriction in the 1- to 11-month arm. *Centre de Santé Integrés* leaders and CHWs expressed the challenges they faced with the age limitation in communities randomly assigned to receive the 1- to 11-month distributions, and all community groups indicated that the 1- to 59-month approach was more acceptable and appropriate for their setting.

The strengths of this study include the cluster-randomized design, which enables an unbiased comparison of the two delivery approaches. In addition, we used implementation science frameworks to define outcomes and their measurement. Both the study team and CHWs were experienced in the intervention approach and data collection methods. Although we attempted to mimic program implementation as closely as possible, by design, some aspects did not accurately reflect operations in a program setting. We used community-level randomization to match the main AVENIR trial and allow for community-level inference across studies. However, programs would plan and implement such a program at the district or CSI level, including all communities within, which distributes supervision costs across more children and thereby lowers the cost per child treated. Additional analyses describing national scale-up costs associated with such implementation are underway. In addition, the predistribution census and post-distribution surveys resulted in communities having more interactions with the team than they might typically have in a program setting, potentially influencing knowledge of the intervention, participation, and acceptability results. The survey questions were developed and refined in close collaboration with Niger team members who speak the relevant local languages to ensure cultural appropriateness and comprehension. However, given the subjective nature of the questions, responses could have been impacted by social desirability bias, are necessarily limited by the questions themselves, and thus may not fully reflect community preferences. The survey was conducted only after the first MDA because of budget limitations, and there may have been important changes in perceptions of acceptability, appropriateness, and feasibility over time that were not captured here. Other limitations include challenges in estimating the target population to accurately measure program reach. Programs typically use administrative estimates of the target population, which are known to be unreliable, especially in Niger, where the last national census was conducted in 2012.^29^^,^^32^^–^^37^ In this study, we used several approaches to estimate treatment coverage and found reasonably consistent results when comparing arms, although caregiver-reported coverage was lower than other estimates, which is a trend often observed in other settings using coverage evaluation surveys to validate administrative coverage estimates.^32^^,^^36^ Overall, we anticipate that our results are generalizable to similar settings in Niger eligible for MDA programs and likely to other similar West African settings.

## CONCLUSION

In this cluster-randomized trial of azithromycin MDA for child survival, we found that including children aged 1–59 months reduced the community cost per dose delivered by an average of $6.50 compared with restricting eligibility to children aged 1–11 months. Overall, community groups perceived both versions of the intervention as acceptable, appropriate, and feasible. Health center leaders, CHWs, and caregivers of eligible children expressed a clear preference for the 1- to 59-month treatment over the 1- to 11-month treatment.

## Supplemental Materials

10.4269/ajtmh.24-0723 Supplemental Materials

## Data Availability

The de-identified dataset and data dictionary will be publicly available at https://osf.io/7vu2p/ at the time of publication.
