# Supplementary material for: Costs, Coverage, and Acceptability of Azithromycin Mass Administration to Children 1–11 Versus 1–59 Months Old to Reduce Mortality: A Cluster-Randomized Trial in Niger
Source: Am J Trop Med Hyg. 2025 Apr 8;112(6):1355–63. doi: 10.4269/ajtmh.24-0723 (PMC12139557; doi:10.4269/ajtmh.24-0723)
Supplement: Supplemental Materials [file tpmd240723.SD1.pdf]

Supplementary Appendix

This appendix has been provided by the authors to give readers additional information about their work.

Supplement to: Arzika AM, Amza A, Alio K et al Costs, coverage, and acceptability of azithromycin mass administration to children 1-11 vs 1-59 months old to reduce mortality: a cluster-randomized trial in Niger

Table of Contents

AVENIR Study Group and Collaborators. 3

Supplementary Table 1. Comparison of community-level treatment coverage outcomes by arm. 5

Supplementary Table 2. Sensitivity analysis of cost per dose delivered examining the difference in average costs among 1-59-month arm versus 1-11-month arm in a linear regression model including a term for population size (N=76). 6

Supplementary Table 3. Estimates of overall costs and costs per dose delivered by treatment arm at the community level, assuming a fixed number of days (2) for delivery. 7

Supplementary Table 4. Detailed costs for delivery and training. 8

Supplementary Table 5. Community health worker survey outcomes by treatment arm. 9

Supplementary Table 6. CSI leader survey outcomes by treatment arm. 10

Supplementary Table 7. Caregiver survey outcomes by arm. 11

Supplementary Table 8. Open-ended responses to the question "Do you know why azithromycin was distributed in your community?" by arm, among community health workers and caregivers, categorized by theme. 12

Supplementary Table 9. Overall perceptions of acceptability, appropriateness and feasibility of 1–59-month vs 1-11 month treatment among community health workers, CSI leaders, and caregivers (N = 76 communities). 13

Supplementary Tables 10. Survey instruments. 14

AVENIR Study Group and Collaborators.

Investigators and Study Personnel

Centre de recherche et interventions en santé publique, Niamey, Niger – Ahmed Arzika, Ramatou Maliki, Bawa Aichatou, Ismael Mamane Bello, Ousseini Boubacar, Nasser Gallo, Karamatoulaye Hamadou, Amadou Harouna, Naser Harouna, Laminou Maliki Haroun, Alio Karamba, Mariama Keimago, Sani Mahamadou, Abarchi Moustapha, Abraham Omar, Farissatou Oumarou

Programme national de santé oculaire, Niamey, Niger – Amza Abdou, Ibrahim Almou, Nassirou Beido, Nouhou Diori Adam, Hamidou Amadou Bagna, Hassane Amadou Traore, Boubacar Kadri, Boubacar Maïdanda, Boubacar Nameywa, Boubacar Mariama, Abba Kaka Hadjia Yakoura, Youssoufou Souley Abdoul Salam

University of California, San Francisco, San Francisco, CA, USA – Benjamin Arnold, Carolyn Brandt, Cindi Chen, Emily Colby, Catherine Cook, Thuy Doan, Jeremy D Keenan, Sandrine Kyane, Victoria Le, Elodie Lebas, Thomas M Lietman, Zijun Liu, William Nguyen, Kieran S O'Brien, Catherine E Oldenburg, Brittany Peterson, Travis C Porco, Kevin Ruder, George W Rutherford, Lina Zhong, Zhaoxia Zhou

Other Study Partners and Collaborators

Bill & Melinda Gates Foundation, Seattle, WA, USA – Rebecca Brander, Dennis Chao, James Heine, Rasa Izadnegahdar, Laura Lamberti, Assaf Oron, Surabhi Rajaram, Matthew Steele

Centre de recherche médical et sanitaire, Niamey, Niger – Ibrahima Issa, Ronan Jambou, Rabiou Labbo, S'Hooshim N. Lamine, Sani Ousmane, Boubakar Rakia, Maikano Sadikou

Centre de santé communautaire, Niamey, Niger – Amadou Zakari

Direction des statistics, Niamey, Niger – Aida Mounkala, Salamatou Maiga, Mahamadou Yahaya

Centre de santé de la mère et de l'enfant, Dosso, Niger – Amina Seyfoulaye

ClinEpiDB, Philadelphia, PA, USA – Brianna Lindsay, Nupur Kittur, David Roos, Sheena Tomko

Comité national éthique pour la recherche en santé, Niamey, Niger – Issa Adji, Djibo Ali, Souleymane Alzouma, Maidanda Boubacar, Diegou Boureima, Cheik Boureima Daouda, Idi Moussa Djatao, Ibrahim Jean Etienne, El Hadji Boubakar H Maiga, Amadou Oumarou, Ocquet Sakina, Sanoussi Samuila

Data and Safety Monitoring Committee – *Emory University, Task Force for Global Health* – David Addiss (chair); *Niger Ministry of Health* – Mourtala Assao; *St. George's University of London* – Julia Bielicki, *Retired from CDC* – Allen Hightower, *Children's Hospital of Colorado* – Brian Jackson, *University of Melbourne* – Fiona Russell

Global Health Strategies, New York, NY, USA – Frances Hocking, Zied Mhirsi, Dan Pawson

International Trachoma Initiative – Paul Emerson, PJ Hooper

Johns Hopkins University, Baltimore, MA, USA – Agbessi Amouzou, Robert Black

Parasites Without Borders, USA – *Charles Knirsch*

Speak Up Africa, Dakar, Senegal – Maelle Ba, Yaye Sophiétou Diop, Yacine Djibo, Gráinne Hutton, Fara Ndiaye

Tampere University, Tampere, Finland **–** *Per Ashorn, Ulla Ashorn*

UCSF-UC Berkeley Center for Global Health Development, Diplomacy, and Economics, CA, USA – Stefano M. Bertozzi, Jim G. Khan, Elliot Marseille

University of Maryland School of Medicine, **Center for Vaccine Development and Global Health,** ***Baltimore, USA*** – Meagan Fitzpatrick

Supplementary Table 1. Comparison of community-level treatment coverage outcomes by arm. Coverage is reported by three different methods (as reported by CHWs which is the primary outcome, using the denominator of eligible children as reported on the pre-distribution census, and using caregiver report). For CHW coverage, estimates are also stratified by sex and age group for each arm.

Analysis	MDA 1		MDA 2		Mean difference (95% CI)[4]	*P*-value[4]
	Azithro 1-59 (n=37)	Azithro 1-11 (n= 39)	Azithro 1-59 (n=37)	Azithro 1-11 (n= 39)		
Overall CHW coverage (mean, SD)[1]	97·7% (3·1%)	93·1% (8·8%)	96·5% (5·2%)	94·0% (7·2%)	2·0% (-1·0%, 4·4%)	0·17
Sex						
Female	97·6% (3·5%)	95·7% (8·2%)	96·3% (6·2%)	94·8% (9·7%)	1·5% (-1·6%, 4·4%)	0·42
Male	97·7% (3·2%)	95·0% (9·0%)	97·0% (4·8%)	94·9% (8·4%)	2·1% (-0·4%, 4·5%)	0·17
Age group						
1-11-months	97·3% (6·0%)	93·1% (8·8%)	95·4% (8·2%)	94·0% (7·2%)	1·3% (-4·9%, 2·2%)	0·45
12-59-months	97.8% (2.8%)	NA	96.8% (5.4%)	NA	NA	NA
Census coverage (mean, SD)[2]	91·6% (11·6%)	91·3% (12·3%)	89·6% (15·4%)	87·8% (17·8%)	1·8% (-3·6%, 7·2%)	0·51
Caregiver-reported coverage (mean, SD)[3]	87·0% (18·0%)	73·8% (23·1%)	NA	NA	13·2% (3·7%, 22·7%)	0·007

CHW, community health worker; CI, confidence interval; MDA, mass drug administration; SD, standard deviation

[1]Number treated and number eligible both from CHW paper data collection form
[2]Number treated from CHW form, number eligible from census
[3]Number treated and number eligible according to caregiver report from post-distribution survey
[4]1-59-month arm compared to 1–11-month arm at MDA 2 for CHW and census coverage, and at MDA 1 for caregiver coverage

Supplementary Table 2. Sensitivity analysis of cost per dose delivered examining the difference in average costs among 1-59-month arm versus 1-11-month arm in a linear regression model including a term for population size (N=76).

Type of cost reported at MDA 2	Unadjusted mean difference (95% CI) [1]	Adjusted mean difference (95% CI) [1,2]	*P*-value
**Delivery cost ($)**	-$4·1 (-$5·3, -$2·9)	-$3·2 (-$4·7, -$1·6)	<0·001
**Training cost ($)**	-$3·6 (-$4·8, -$2·6)	-$2·8 (-$4·2, -$1·4)	<0·001
**Delivery + training cost ($)**	-$7·7 (-$10·1, -$5·4)	-$6·0 (-$8·9, -$3·1)	<0·001

CI, confidence interval; MDA, mass drug administration

[1]1–59-month azithro arm compared to 1–11-month azithro arm at MDA 2 (REF)
[2]Adjusted for 1–59-month-old population size from the census

Supplementary Table 3. Estimates of overall costs and costs per dose delivered by treatment arm at the community level, assuming a fixed number of days (2) for delivery.

Variable	MDA 1		MDA 2		Mean difference in MDA 2[1] (95% CI)	*P*-value[1]
	Azithro 1-59 (n=37)	Azithro 1-11 (n= 39)	Azithro 1-59 (n=37)	Azithro 1-11 (n= 39)		
**Overall costs and doses delivered**						
Delivery cost (geometric mean, 95% CI)	$125·1 ($124·7, $125·4)	$110·6 ($110·3, $111·0)	$127·9, ($127·6, $128·3)	$113·4 ($113·0, $113·7)	$14.6 ($10.2, $19.1)	<0·0001
Training cost (geometric mean, 95% CI)	$136·8 ($136·4, $137·1)	$143·9 ($143·6, $144·3)	$81·5 ($81·2, $81·8)	$81·2 ($80·9, $81·6)	$0·3 (NA)[2]	<0·0001
Delivery + training cost (geometric mean, 95% CI)	$262·0 ($261·6, $262·4)	$254·6 ($254·3, $254·9)	$209·7 ($209·3, $210·0)	$194·7 ($194·3, $195·0)	$15.0 ($10.4, $19.7)	<0·0001
Doses delivered (geometric mean, SD)	126·17 (85·5)	24·6 (14·0)	157·5 (119·7)	25·7 (15·3)	131·8 (91·6, 172·0)	<0·0001
**Cost per dose delivered**						
Delivery cost (geometric mean, 95% CI)	$1·2 ($0·6, $1·8)	$5·3 ($4·7, $5·8)	$1·1 ($0·4, $1·7)	$5·2 ($4·7, $5·8)	-$4.2 (-$5.2, -$3.3)	<0·0001
Training cost (geometric mean, 95% CI)	$1·3 ($0·7, $2·0)	$6·8 ($6·3, $7·4)	$0·7 ($0·0, $1·4)	$3·8 ($3·2, $4·4)	-$3·1 (-$3·9, -$2·4)	<0·0001
Delivery + training cost (geometric mean, 95% CI)	$ 2·6 ($1·9, $3·2)	$12·1 ($11·5, $12·7)	$1·7 ($1·1, $2·4)	$9·0 ($8·4, $9·6)	-$7.3 (-$9.1, -$5.7)	<0·0001

CI, confidence interval; MDA, mass drug administration

[1]1-59-month arm compared to 1-11-month arm at MDA 2

Supplementary Table 4. Detailed costs for delivery and training.

**Cost categories**	**MDA**	**Cost (in USD)**
**Delivery costs**		
Base costs		
Community health worker (Relais) payment	1 and 2	22
Equipment		
Mineral water	1 and 2	6.2
Syringes	1 and 2	1.7
Dosing cups	1 and 2	1.7
Backpacks	1 and 2	11
Mobilizer guide payments	1 and 2	0.63
Payment to chief	1 and 2	9.1
Personnel costs within each delivery method arm		
CSI Chief per diem (Supervision)	1 and 2	18.18
CSI Chief Communication	1 and 2	4.55
Case agent supervision	1 and 2	12.73
District agent supervision	1 and 2	18.18
**Training costs** *within each delivery method arm*		
AVENIR Supervisor per diem	1 and 2	36.36
Relais per diem	1 and 2	9.09
Relais Transportation	1 and 2	5.45
Relais Meals and Coffee	1 and 2	4.55
Relais Notebooks and Pens	1 and 2	2.73
CSI Chief per diem	1	36.36
CSI Chief per diem	1 and 2	18.18
CSI Chief Meals and Coffee	1	9.09
CSI Chief Meals and Coffee	1 and 2	4.55
CSI Chief Transportation	1	14.55
Room Rental	1	90.91
District Chief per diem	1	54.55
District Chief Meals and Coffee	1	9.09
District Chief Communication	1 and 2	36.36
CSI Chief Communication	1 and 2	4.55
District Communicator Communication	1	18.18
District Communicator Communication	1 and 2	18.18
Case agent per diem	1 and 2	12.73
Case agent meals and coffee	1 and 2	4.55
Case agent transportation	1 and 2	5.45

CSI, Centre de Santé Integré; MDA, mass drug administration; USD, US dollars

Supplementary Table 5. Community health worker survey outcomes by treatment arm.

Survey question	Azithro 1-59 (n=37) Mean % responding "Yes" (95% CI)	Azithro 1-11 (n=39) Mean % responding "Yes" (95% CI)	Mean difference (95% CI)[1]	*P*-value[1]
Do you know why azithromycin was distributed in your community?	86·4% (74·4%,98·5%)	92·8% (83·0%,102·6%)	-6·4% (-21·5%, 8·8%)	0·44
Have you reported any side effects?	6·5% (-1·3%,14·3%)	1·3% (-0·6%, 3·2%)	5·2% (-2·8%, 13·2%)	0·32
Did you encounter:				
Guardians and children from non-target communities present for treatment	50·4% (32·9%, 67·9%)	36·6% (16·1%, 57·1%)	13·8% (-12·5%, 40·1%)	0·29
Guardians requested ineligible children be treated	50·6% (33·5%, 67·5)	49·4% (29·8%, 69·0%)	1·2% (-24·1%, 26·6%)	0·93
Guardians pretended ineligible children were eligible for treatment	46·4% (29·4%, 63·3%)	52·9% (32·9%, 72·8%)	-6·5% (-32·1%, 19·0%)	0·61
Guardians presented the same child multiple times for treatment	15·8% (2·2%, 29·3%)	8·83% (-2·7%, 20·3%)	6·9% (-10·4%, 24·2%)	0·44
Do you prefer the program distributes to:				
1-11 months	15·1% (4·5%, 25·7%)	14·0% (0·6%, 27·4%)	1·1% (-15·5%, 17·8%)	0·89
1-59 months	84·9% (74·3%, 95·5%)	86·1% (72·6%, 99·5%)	-1·1% (-17·8%, 15·5%)	0·89
Is the program more appropriate for you if it distributes to:				
1-11 months	19·4% (6·5%, 32·3%)	18·5% (2·9%, 34·1%)	0·9% (-18·8%, 20·6%)	0·93
1-59 months	80·6% (67·7%, 93·5%)	81·5% (65·9%, 97·1%)	-0·9% (-20·6%, 18·8%)	0·93
Is the program more feasible for you if it distributes to:				
1-11 months	16·7% (5·0%, 28·4%)	13·7% (0·6%, 26·8%)	3·0% (-14·1%, 20·1%)	0·74
1-59 months	83·3% (71·6%, 95·0%)	86·3% (73·2%, 99·4%)	-3·0% (-20·1%, 14·1%)	0·73

CI, confidence interval

[1]1-59-month arm compared to 1–11-month arm at post-MDA 1 survey

Supplementary Table 6. CSI leader survey outcomes by treatment arm.

Survey question	Azithro 1-59 (n=37) Mean % responding "Yes" (95% CI)	Azithro 1-11 (n=39) Mean % responding "Yes" (95% CI)	Mean difference (95% CI)[1]	*P*-value
Do you know why azithromycin was distributed to communities associated with your health center?	100%	100%	0·0	1·00
Do the CHWs bring up the difficulties encountered in relation to the intervention?	14·3% (-2·0%, 30·6%)	3·5% (-3·6%,10·5%)	10·8% (-6·7%, 28·4%)	0·30
Do you prefer the program distributes to 1-11 months or 1-59 months?				
1-11 months	19·1% (0·7%, 37·4%)	29·9% (12·5%, 47·3%)	-10·8% (-35·4%, 13·8%)	0·51
1-59 months	81·0% (62·6%, 99·3%)	63·2% (44·9%, 81·6%)	17·7% (-7·5%, 43·0%)	0·22
Neither	0·0%	6·9% (-2·9%,1 6·7%)	-6·9% (-16·7%, 2·9%)	0·51
Is the program more appropriate for your community if it distributes to 1-11 months or 1-59 months?				
1-11 months	19·1% (0·7%, 37·4%)	14·9% (1·6%, 28·3%)	4·1% (-18·0%, 26·2%)	0·88
1-59 months	81·0% (62·6%, 99·4%)	78·2% (62·5%, 93·8%)	2·8% (-20·7%, 26·2%)	0·88
Neither	0·0%	6·9% (-2·9%,16·7%)	-6·9% (-16·7%, 2·9%)	0·50
Is the program more feasible for your community if it distributes to 1-11 or 1-59 months?				
1-11 months	14·3% (-2·0%, 30·6%)	21·8% (6·2%, 37·5%)	-7·6% (-29·6%, 14·4%)	0·60
1-59 months	85·7% (69·4%,102·0%)	71·3% (54·1%, 88·5%)	14·5% (-8·6%, 37·5%)	0·25
Neither	0·0%	6·9% (-2·9%, 16·7%)	-6·9% (-16·7%, 2·9%)	0·51
The distribution of azithromycin reduces mortality (yes %)	100%	100%	0·0	1·00
Do you keep a register of the children in this CSI? (yes%)	61·9% (39·3%, 84·6%)	48·3% (28·9%, 67·6%)	13·6% (-15·4%, 42·6%)	0·40

CHW, community health worker; CI, confidence interval; CSI, Centre de Santé Integré (primary health care center)

[1]1-59-month arm compared to 1–11-month arm at post-MDA 1 survey

Supplementary Table 7. Caregiver survey outcomes by arm.

Survey question	Azithro 1-59 (n=37) Mean % responding "Yes" (95% CI)	Azithro 1-11 (n=39) Mean % responding "Yes" (95% CI)	Mean difference (95% CI)[1]	*P*-value
Do you know why azithromycin was distributed in your community?	69·5% (59·8%, 79·2%)	81·5% (73·2%, 89·8%)	-12·0% (-24·5%, 0·6%)	0·06
I think this intervention is desirable for our household	98·8% (97·9% 99·7%)	94·6% (90·5%,98·7%)	4·2% (0·0%, 8·4%)	0·04
I think this intervention is appropriate for our household	98.9% (98.0%, 99.8%)	95.4% (92.2%, 98.7%)	3·4% (0·1%, 6·8%)	0·04
I think this intervention is feasible for our household	99·0% (98·3%, 99·7%)	96·5% (93·6%,99·4%)	2·5% (-0·5%, 5·4%)	0·08
How important is this intervention to you compared to other child health interventions of children less than 5 years old in your community? (1-5)	4·0 (3·8, 4·1)	4·1 (3·9, 4·3)	-0·1 (-0·3, 0·1)	0·26
Do you prefer the program distributes to:				
1-11 months	17·1% (9·8%, 24·4%)	21·9% (14·1%, 29·7%)	-4·8% (-15·3%, 5·6%)	0·38
1-59 months	82·9% (75·6%, 90·2%)	78·1% (70·3%, 85·9%)	4·8% (-5·6%, 15·3%)	0·37
Is the program more appropriate for your household if it distributes to:				
1-11 months	14·4% (7·6%, 21·1%)	20·7% (13·2%, 28·1%)	-6·3% (-16·2%, 3·6%)	0·21
1-59 months	85·6% (78·8%, 92·4%)	79·3% (71·8%, 86·8%)	6·3% (-3·6%, 16·2%)	0·21
Is the program more feasible for your household if it distributes to:				
1-11 months	18·7% (10·3%, 27·2%)	20·0% (12·5%, 27·5%)	-1·3% (-12·4%, 9·8%)	0·82
1-59 months	81·3% (72·8%, 89·7%)	80·0% (72·6%, 87·5%)	1·3% (-9·8%, 12·4%)	0·82

CI, confidence interval

[1]1-59-month arm compared to 1–11-month arm at post-MDA 1 survey

Supplementary Table 8. Open-ended responses to the question "Do you know why azithromycin was distributed in your community?" by arm, among community health workers and caregivers, categorized by theme.

	Azithro 1-11 month<br>N (%)	Azithro 1-59 month<br>N (%)	Total<br>N (%)
**Community health worker responses (n=67)**			
Fight against diseases	10 (29.4%)	19 (55.9%)	29 (43.3%)
Reduce mortality	8 (23.5%)	10 (29.4%)	18 (26.9%)
Fight trachoma/eye diseases	3 (8.8%)	3 (8.8%)	6 (8.9%)
Promote child health	2 (5.9%)	3 (8.8%)	5 (7.5%)
Other	14 (41.2%)	0 (0%)	14 (20.9%)
**Caregiver responses (n=2,588)**			
Fight against diseases	343 (26.5%)	373 (28.8%)	716 (27.7%)
Reduce mortality	497 (38.3%)	610 (47.0%)	1107 (42.8%)
Fight trachoma/eye diseases	112 (8.6%)	163 (12.6%)	275 (10.6%)
Promote child health	82 (6.3%)	86 (6.6%)	168 (6.5%)
Prevention	107 (8.3%)	205 (15.8%)	312 (12.1%)
Good antibiotic	43 (3.3%)	12 (0.9%)	55 (2.1%)
Other	57 (4.4%)	82 (6.32%)	139 (5.4%)

Supplementary Table 9. Overall perceptions of acceptability, appropriateness and feasibility of 1–59-month vs 1-11 month treatment among community health workers, CSI leaders, and caregivers (N = 76 communities). Note that this table does not display a by arm comparison; rather, this table shows the stated preference for 1-59- or 1-11-month treatment by stakeholder group in both arms combined. The information displayed here corresponds to that presented in Figure 2.

Outcome and Stakeholder Group	Prefer Azithro 1-59	Prefer Azithro 1-11	Mean difference (95% CI)[1]	*P*-value
Acceptability (affective attitude)				
CHWs	85·4% (77·3%, 93·4%)	14·6% (6·6%, 22·7%)	70·8% (59·5%, 82·1%)	<0·0001
CSI leader	70·7% (57·7%, 83·6%)	25·3% (13·0%, 37·7%)	45·3% (27·7% 63·0%)	<0·0001
Caregivers	80·4% (75·2%, 85·6%)	19·6% (14·4%, 24·9%)	60·8% (53·4%, 68·1%)	<0·0001
Appropriateness				
CHWs	81·0% (71·4%, 90·6%)	19·0% (9·4%, 28·6%)	62·0% (48·6%, 75·4%)	<0·0001
CSI leader	79·3% (67·9%-90·8%)	16·7% (6·1%, 27·2%)	62·7% (47·3%, 78·0%)	<0·0001
Caregivers	82·4% (77·4%, 87·4%)	17·6% (12·7%, 22·6%)	64·7% (57·7%, 71·7%)	<0·0001
Feasibility				
CHWs	84·6% (76·2%, 93·0%)	15·4% (7·0%, 23·8%)	69·2% (57·4%, 80·9%)	<0·0001
CSI leader	77·3% (65·5%, 89·2%)	18·7% (7·7%, 29·7%)	58·7% (42·7%, 74·7%)	<0·0001
Caregivers	80·6% (75·1%, 86·1%)	19·4% (13·9%, 24·9%)	61·2% (53·6%, 68·9%)	<0·0001

CHW, community health worker; CI, confidence interval; CSI, Centre de Santé Integré (primary health care center)

[1]Preference for 1-59-month compared to 1–11-month delivery approach as assessed during the post-MDA 1 survey

Supplementary Tables 10. Survey instruments. Survey questions are presented in English below. Survey instruments were developed in English then translated to French. The French translations were reviewed as a group by study team members fluent in English and French as well as local languages, including Zarma, Hausa and Peul. The final wording for each question was decided based on group consensus. For acceptability, appropriateness, and feasibility, English and French translations of the AIM, IAM, and IFM validated instruments were reviewed by the group to determine the appropriate wording in each language for the single question used to reflect the domain. In addition, training sessions involved discussions of translations of each question to each relevant local language, including Zarma, Hausa, and Peul to ensure comprehensibility.

1. **Local Health Center Leaders**

Question	Response options	Outcome
Do you know why azithromycin was distributed to communities associated with your health center? If so, why?	Two questions – Yes/No for the first, open-ended text field for the second if Yes (don't prompt with response options)	Fidelity<br>Acceptability - coherence
Do the CHWs bring up the difficulties encountered in relation to the intervention?	Two questions – Yes/No for the first, open-ended text field for the second if Yes (don't prompt with response options)	Fidelity<br>Acceptability - burden
Consider a program that distributes azithromycin to only children 1-11 months of age compared to a program that distributes to all children 1-59 months of age.<br>1. Do you prefer the program distributes to 1-11 months or 1-59 months?<br>2. Is the program more appropriate for your community (or your household, for the guardian questionnaire) if it distributes to 1-11 months or 1-59 months?<br>3. Is the program more feasible for your community (or you for the CHW questionnaire, or for the household for the guardian questionnaire) if it distributes to 1-11 or 1-59 months?	• 1-11, 1-59, and maybe neither<br>• Yes/No for each, open-ended text field for each option selected «Please specify why»<br>• Yes/No for each, open-ended text field for each option selected «Please specify why»	Acceptability – affective attitude (1)<br>Appropriateness (2)<br>Feasibility (3)
Do you keep a register of the children in this health center?<br>If yes, text fields to enter # children 1-11 months of age, # children 1-59 months of age?	Two questions – Yes/No for the first, then two number text fields to enter # 1-11 and # 1-59	Reach

2. **Community health workers**

Question	Response options	Outcome
Do you know why azithromycin was distributed in your community? If so, why?	Two questions – Yes/No for the first, open-ended text field for the second if Yes (don't prompt with response options)	Fidelity<br>Acceptability - coherence

Have you reported any side effects? If yes how much?	Two questions – Yes/No for the first, open-ended text field for the second if Yes (don't prompt with response options)	Acceptability
Did you encounter any of the following?<br>• Guardians and children from non-target communities presented for treatment<br>• Guardians requested ineligible children be treated (ex: children out of age range)<br>• Guardians pretended ineligible children were eligible for treatment\<br>• Guardians presented the same child multiple times for treatment<br>• Other (please specify):	Yes/No for each	Reach<br>Acceptability
Consider a program that distributes azithromycin to only children 1-11 months of age compared to a program that distributes to all children 1-59 months of age.<br>1. Distributing to 1-11 months only is more/less desirable than distributing to 1-59 months (*acceptability*)<br>2. Distributing to 1-11 months only is more/less appropriate than distributing to children 1-59 months (*appropriateness*)<br>3. Distributing to 1-11 months only is more/less feasible than distributing to children 1-59 months of age (*feasibility*)	Yes/No for each, open-ended text field for each option selected «Please specify why»	Acceptability – affective attitude (1)<br>Appropriateness (2)<br>Feasibility (3)
Do you keep a register of the children in this community?<br>If yes, text fields to enter # children 1-11 months of age, # children 1-59 months of age?	Two questions – Yes/No for the first, then two number text fields to enter # 1-11 and # 1-59	Reach/penetration

3. Guardians

**Question**	**Response options**	**Outcome**
Do you know why azithromycin was distributed in your community? If so, why?	Two questions – Yes/No for the first, open-ended text field for the second if Yes (don't prompt with response options)	Fidelity<br>Acceptability - coherence
Did you participate in this program by having your child treated with azithromycin? If yes, why? If not, why not?	Two questions – Yes/No for the first, open-ended text field for the second if Yes (don't prompt with response options)	Reach

Did the child had any side effects after treatment such as nausea, vomiting, diarrhea, abdominal pain, skin rash and other?	Follow-up text field as response options	Acceptability – burden
For the following statements, tell me if you agree or disagree:<br><br>1. I think this intervention is desirable for our household<br>2. I think this intervention is appropriate for our household<br>3. I think this intervention is feasible for our household	Yes/No for each, open-ended text field for each option selected «Please specify why»	Acceptability – affective attitude (1)<br>Appropriateness (2)<br>Feasibility (3)
How important is this intervention to you compared to other child health interventions of children less than 5 years old in your community?	• Least important (*)<br>• Less important (**)<br>• Same importance (***)<br>• More important (****)<br>• Most important (*****)<br><br>Shown with a visual scale	Relative priority
Consider a program that distributes azithromycin to only children 1-11 months of age compared to a program that distributes to all children 1-59 months of age.<br>1. Distributing to 1-11 months only is more/less desirable than distributing to 1-59 months (*acceptability*)<br>2. Distributing to 1-11 months only is more/less appropriate than distributing to children 1-59 months (*appropriateness*)<br>3. Distributing to 1-11 months only is more/less feasible than distributing to children 1-59 months of age (*feasibility*)	Yes/No for each, open-ended text field for each option selected «Please specify why»	Acceptability – affective attitude (1)<br>Appropriateness (2)<br>Feasibility (3)

Summary of implementation outcomes and constructs

- **Acceptability.** Perception that the intervention is agreeable, palatable, or satisfactory.[1,2]
  - **Affective attitude.** How an individual feels about the intervention[4]
  - **Coherence.** The extent to which the participant understands the intervention and how it works.[4]
  - **Burden.** The perceived amount of effort that is required to participate in this intervention.[4]
    - Note that the final version of the survey didn't quite directly capture this, consider removing.
- **Appropriateness.** Perceived fit, relevance, or compatibility of the intervention.[1,2]
- **Feasibility.** Perception of the extent to which the intervention can successfully be carried out.[1,2]
- **Fidelity.** Degree to which the intervention was implemented according to the protocol.[1]
  - The acceptability-coherence questions overlap with the survey-based measure of fidelity, since understanding the intervention is an aim of the protocol
- **Reach.**[3] Individual-level measure of participation in the intervention.
  - Note that the survey-based indicator of participation is one of several measures of Reach included in this study.
- **Relative priority.**[5] Perception of importance of the intervention with respect to other similar interventions.

References

- **Implementation outcomes defined with guidance from**
    - [1]Proctor E, Silmere H, Raghavan R, Hovmand P, Aarons G, Bunger A, Griffey R, Hensley M. Outcomes for implementation research: conceptual distinctions, measurement challenges, and research agenda. Adm Policy Ment Health. 2011 Mar;38(2):65-76. doi: 10.1007/s10488-010-0319-7. PMID: 20957426; PMCID: PMC3068522.
    - [2]Weiner BJ, Lewis CC, Stanick C, Powell BJ, Dorsey CN, Clary AS, Boynton MH, Halko H, 2017. Psychometric assessment of three newly developed implementation outcome measures. Implementation Science 12: 108.
    - [3]Glasgow RE, Vogt TM, Boles SM. Evaluating the public health impact of health promotion interventions: the RE-AIM framework. Am J Public Health. 1999 Sep;89(9):1322-7. doi: 10.2105/ajph.89.9.1322. PMID: 10474547; PMCID: PMC1508772.

- **Acceptability constructs defined with guidance from**
    - [4]Sekhon, M., Cartwright, M. & Francis, J.J. Acceptability of healthcare interventions: an overview of reviews and development of a theoretical framework. *BMC Health Serv Res* **17**, 88 (2017). https://doi.org/10.1186/s12913-017-2031-8

- **Relative priority defined with guidance from**
    - [5]Consolidated Framework for Implementation Research (CFIR). https://cfirguide.org/constructs-old/relative-priority/
